# Climate-Altered Wetlands Challenge Waterbird Use and Migratory Connectivity in Arid Landscapes

**DOI:** 10.1038/s41598-019-41135-y

**Published:** 2019-03-15

**Authors:** Susan M. Haig, Sean P. Murphy, John H. Matthews, Ivan Arismendi, Mohammad Safeeq

**Affiliations:** 1 U.S. Geological Survey, Forest and Rangeland Ecosystem Science Center, Corvallis, Oregon USA; 2 Present Address: Pennsylvania Game Commission, Harrisburg, Pennsylvania USA; 3 Alliance for Global Water Adaptation, Corvallis, Oregon USA; 4 Department of Fisheries and Wildlife, Oregon State University, Corvallis, Oregon USA; 5 Sierra Nevada Research Institute, University of California, Merced, California USA

## Abstract

Wetlands in arid landscapes provide critical habitat for millions of migratory waterbirds across the world and throughout their annual cycle. The scope and scale of understanding avian use of these wetlands in conjunction with changes in climate are daunting yet critical to address lest we lose continent-wide migratory pathways. Here, we assess changes in waterbird use of North America’s Pacific Flyway in the Great Basin by examining water availability and climate trends over the past 100 years. We found recent (1980–2015) climate warming has significantly reduced the amount and shifted seasonality of water flowing into wetlands. Further, we found remarkable changes in waterbird species composition over time. We propose that a reduced hydroperiod and lower water quality from reduction in water level and flow limits sites used by waterbirds. These factors reduce chick survivorship as they cannot metabolize saline water, which makes suitable freshwater conditions a limiting resource. Collectively, climate-induced changes in Great Basin wetlands suggest a major shift in freshwater ecosystems, resulting in degradation of a continental migratory route. This work illustrates the importance of examining multi-scale changes in critical regional resources to understand their impact across a hemispheric flyway and provides a model to examine other flyways.

## Introduction

Understanding changes in environmental conditions at multiple spatial and temporal scales is essential for regional habitat conservation planning and for evaluating the status of hemispheric migratory pathways^1,2^. Species that migrate long distances require a diversity of resources throughout their annual cycle to survive and reproduce^1–3^. Yet recent evidence suggests that just 9% of 1451 migratory bird species are adequately covered by protected areas across all stages of their annual cycle^2^.

Recent climate shifts increase the risks of mismatches between species phenology and environmental conditions resulting in deterioration of major migratory pathways^4–8^. From the disappearance of salt pans in the Mediterranean region to degradation of migratory stopover sites in the North American prairie pothole region and the Mohave desert, migratory waterbirds (e.g., waterfowl, shorebirds, wading birds) and other avian species are undergoing significant population declines due to large-scale climate-induced habitat changes^7–9^.

**Figure 1:**
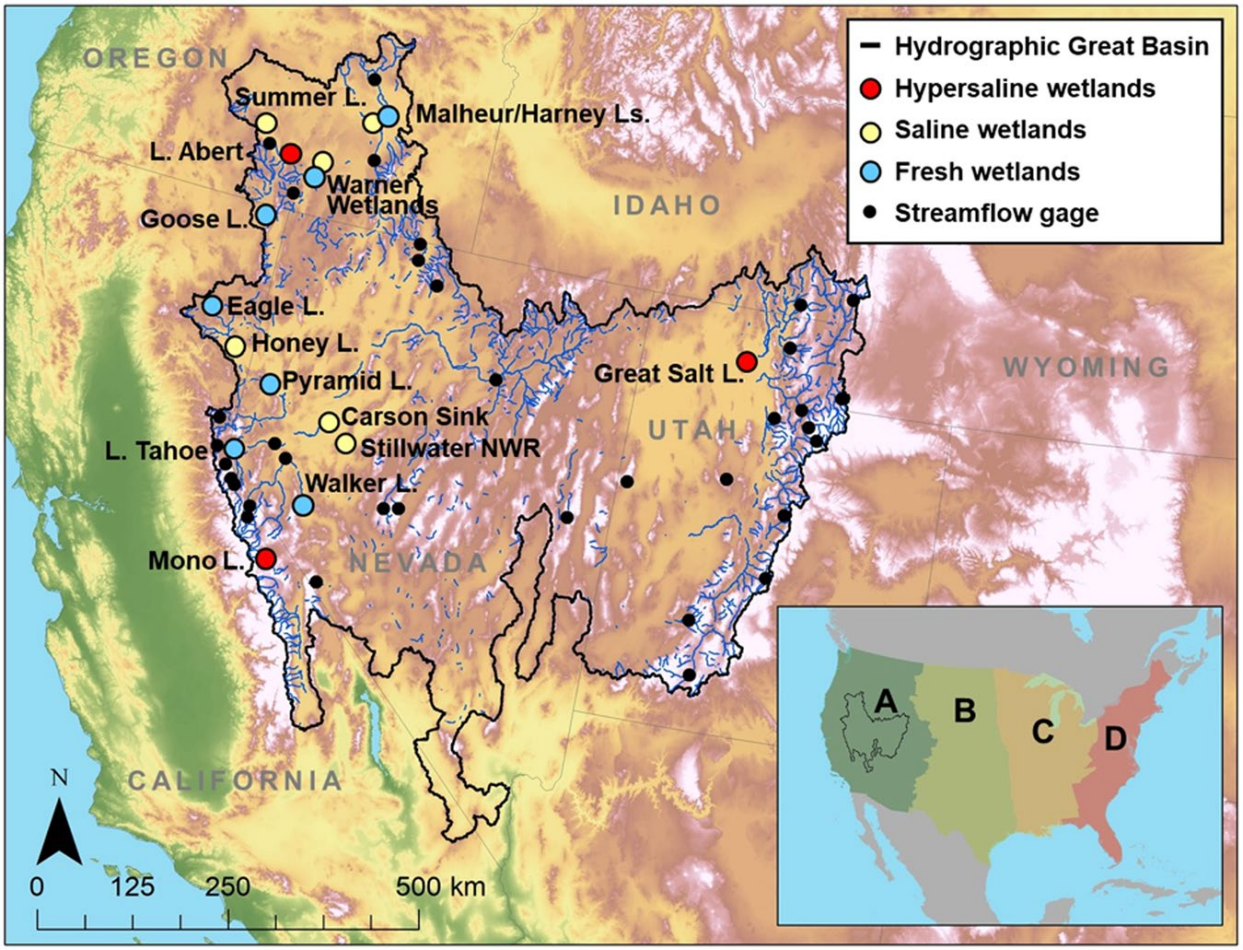
Major Great Basin wetlands (and their associated salinities) used by millions of waterbirds throughout the annual cycle. North America’s three hypersaline lakes (i.e., Lake Abert, Mono Lake and Great Salt Lake) occur in the Great Basin. Inset map shows U.S. Fish and Wildlife Service North American migratory bird flyways: (**A**) Pacific, (**B**) Central, (**C**) Mississippi and (**D**) Atlantic. The map does not extend into Canada as flyways mix in the north.

**Figure 2:**
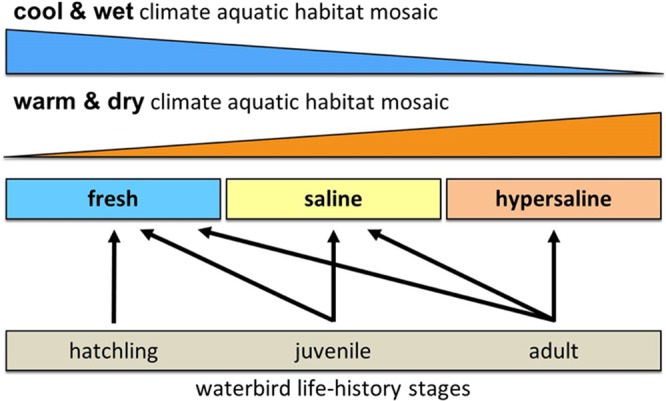
Hydro-climatic relationships in dry systems. Associations of climate variability with wetland water type (fresh, saline and hypersaline) and the connection to specific migratory waterbird life-history stages. The relationship between wetland type and climate illustrates the contraction of variability in wetland type during wet (blue wedge) and dry (orange wedge) years. As shifts continue toward a warmer, drier climate, the diversity of wetland types will transform.

Arctic and sub-Arctic waterbirds typically follow four migratory flyways in North America (Pacific, Central, Mississippi and Atlantic) on their way to and from breeding and wintering sites (Fig. 1). In the western U.S., millions of migratory waterbirds follow the Pacific migratory flyway through a mosaic of Great Basin wetlands (Fig. 1)^9–11^. The Great Basin wetland matrix nearly spans the width of the Pacific Flyway, so birds using the western interior of North America are likely to interact with the Great Basin landscape. A major attraction for migrants is the abundance of aquatic invertebrates in saline and hypersaline wetlands^9–12^. The hypersaline lakes of the Great Basin provide a rich food source and have been identified as hemispherically important stopover sites. In fact, many migratory waterbirds follow a latitudinal path connecting two or three of these hypersaline lakes during their migration (Fig. 1)^12^. Conversely, newly-hatched waterbirds must be raised near fresh water as they do not yet have a well-developed salt gland to cope with heavy salt loads (Fig. 2)^13–19^. Thus, waterbird use of these arid-land wetlands during any phase of the annual cycle depends on physiological adaptations that take advantage of abundant saline wetland-derived prey that link metabolism, digestion, and osmosis^19^.

Great Basin wetlands sustain avian populations during the all phases of the annual cycle and would be impossible to replace if the matrix of freshwater wetlands, saline lakes and invertebrate resources were lost due to climate change or other processes. During spring migration, 70% (over 2 million birds) of Pacific Flyway waterfowl pass through the Southern Oregon-Northeastern California (SONEC) region^9–11^. Peak migration counts for shorebirds include 1.5 million at the Great Salt Lake, 214,000 in Lahontan Valley, CA, 102,000 at Mono Lake, CA and 83,000 at Lake Abert, OR. Without alternative breeding or migration sites to absorb these birds, bypassing the Great Basin on migration and moving further south would not be energetically possible. This is particularly true as the next point south would be the Salton Sea in southern California (approximately 600 km from Mono Lake), where water recently has been diverted^20,21^.

These wetlands are key for significant portions of individual species. For example, the entire North American population of Eared Grebes (*Podiceps nigricollis*), up to 90% of all Wilson’s Phalaropes (*Phalaropus tricolor*) and over 50% of American Avocets (*Recurvirostra americana*) stop at these sites. Large colonies of American White Pelicans (*Pelecanus erythrorhynchos*) (up to 50% of the global population), American Avocets and Western Snowy Plovers also breed in the Great Basin^9,10,22^.

Extensive evidence already indicates regional declines in Great Basin waterbird abundance^9,10,22,23^. Great Basin shorebird populations have experienced a 70% decline since 1973, and more than half of the nine most important western saline lakes for birds have diminished by 50–95% over the past 150 years. Among nine Great Basin breeding waterbirds considered, Langham *et al*.^24^ found seven [i.e., Eared Grebe, American White Pelican, White-faced Ibis (*Plegadis chihi*), American Avocet, Snowy Plover (*Charadrius nivosus*), Marbled Godwit (*Limosa fedoa*), and Wilson’s Phalarope] to be in danger of losing half or more of their current range by 2050 due to climate change.

These seven Great Basin breeding waterbirds are migratory and risks to their success may occur in any phase of the annual cycle, with effects often carried over to the next phase^1^. However, these species generally do not migrate long distances, often preferring to winter further south in the Great Basin, Salton Sea, or northern Mexico^22^. Thus, they face some of the same limitations during other phases of the annual cycle^25^. For example, increased salinity due to decreased inflows at the Salton Sea has reduced the density and diversity of fish and invertebrate prey for waterbirds^20,21,26^. Not only has this resulted in a decline of birds using this important site, but it is not clear where the displaced species are finding suitable habitat.

Species that use the Great Basin only as short-term migration habitat may encounter different risks, positive or negative, as a result of altered habitats, prey abundance, predator pressure or human interference in their Arctic breeding or tropical winter areas. Thus, species’ needs must be viewed across the annual cycle and full migratory pathway to gain a balanced perspective of the influence of climate change or other factors on individual survivorship or population trajectories.

The regional wetland mosaic supporting fresh and saline systems in the Great Basin depends on the hydro-climatic envelope enabling these distinct habitat-types to exist in close proximity (Fig. 1). For internally draining (endorheic), seasonally variable water systems like those in the Great Basin, paleo-ecological evidence suggests that hydrologic systems have made dramatic shifts since the mid-Holocene (ca. 7,000 years BP)^27,28^. Since that time, the Great Basin has transformed from a predominately large perennial freshwater riparian system connected to the Pacific Ocean drainage into a matrix of increasingly isolated freshwater, saline (3–34% salt concentration) and hypersaline lakes (>35% salt concentration), wetlands and small seeps (Fig. 1). Thus, wetland community composition is largely determined by salinity in the Great Basin^29–31^. Climate change may be driving impacts comparable in scale over coming decades.

Indeed, paleoclimatology shows Great Basin wetland systems are inherently vulnerable to even minor climatic shifts. Their hydrological inputs derive primarily from precipitation (rain, snowpack) and lose water through evapotranspiration^23,29,32–39^. Recent changes in water availability, quality (defined as salinity) and quantity have altered the viability of these critical breeding sites and migratory pathways^40–43^. In the future, warmer winter and spring seasons driving snowmelt patterns are predicted to shift peak flows to earlier in the year as more precipitation will fall as rain instead of snow^38^.

In this report, we consider multi-scale changes in hydro-climatology across the Great Basin and examine subsequent effects on waterbird needs, community composition and waterbird migratory connectivity using over 100 years of spatially explicit temperature and precipitation data, trends in streamflow^44^ and recent trends in Breeding Bird Survey (BBS) data^45^. Previous studies have explored the association between climate-driven shifts in temperature and precipitation regimes^46,47^, yet few studies have made the direct link between climate change impacts on environmental water quantity and quality and subsequent biological responses^7,23,48^. We demonstrate that climate-induced increasing temperatures and shortened water seasons can have a negative effect on waterbird use and productivity among inland, arid land wetlands. Regional water management strategies will need to consider the mosaic of water needs throughout the annual cycle in order to forestall loss of critical breeding grounds and a hemispheric migratory pathway.

## Results

### Hydro-climatic changes result in reduced water availability and shorter hydroperiod

**Figure 3:**
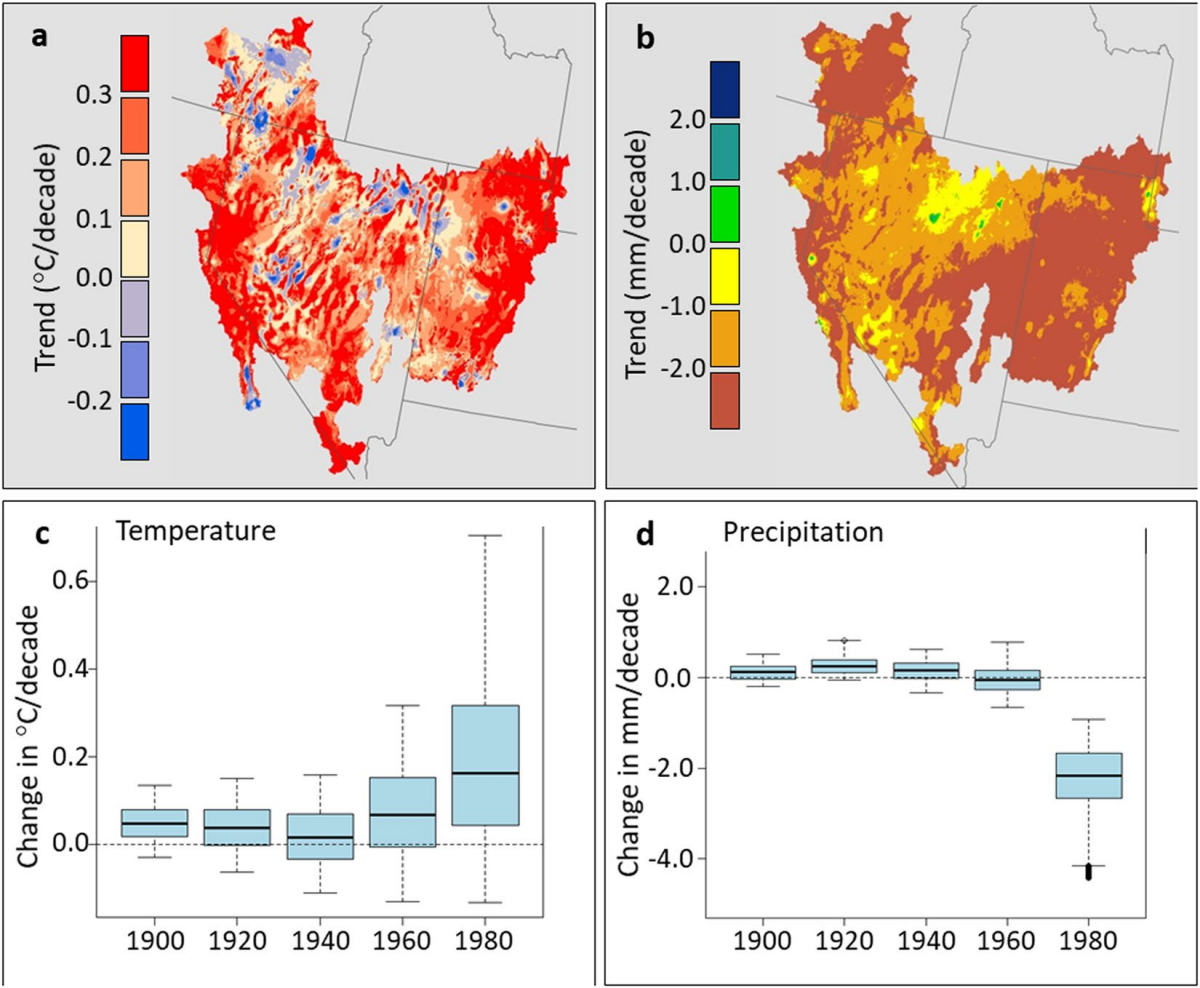
Great Basin climate trends. (**a**) Spatial trends in annual minimum air temperature °C/decade) and (**b**) total annual precipitation (mm/decade) from 1980–2008. (**c**) Distribution of air temperature trends and (**d**) precipitation beginning with 1900–2008 and ending with 1980–2008.

We found mean annual minimum air temperatures increased in the Great Basin over the past century or more (1900–2008: mean = 0.09 °C/decade, range = 0.01 to +0.17, Fig. 3, Supplementary Figs S1–6). Moreover, rates of change in annual air temperature during the period 1980–2008 increased more than twofold (Fig. 3; mean = 0.23 °C/decade, range = −0.05 to +0.62) compared to that of the long-term trend (1900–2008). The greatest seasonal increase in air temperatures occurred during the summers (July-September) from 1980–2008 (mean = 0.16 °C/decade, range = −0.13 to +0.71), affecting more than half of the Great Basin area (Supplementary Fig. S6).

In the Great Basin, annual and seasonal precipitation has only changed marginally over the past century (mean + 1.15 mm/decade; range −2.03 to +5.16). However, during the most recent period (1980–2008) the majority of the region showed trends toward a drier climate (Fig. 3, Supplementary Fig. S1). Further, streamflow trends indicate that shifting precipitation from more snow to rain in winter resulted in an earlier arrival and a consistent decline in water availability (Supplementary Table S1). During this period, most quantiles of annual flow occurred earlier (1–2 days/decade) in the water year. Consequently, the area has experienced a reduction in the duration of the hydroperiod (i.e., difference between timing of the 5^th^ to 95^th^ percentile of the annual discharge) of approximately one day per decade (Supplementary Table S1). These trends were consistent throughout the entire region, as indicated by our results using the regional Mann-Kendall test^49,50^.

### Shifts in waterbird biodiversity over time

**Figure 4:**
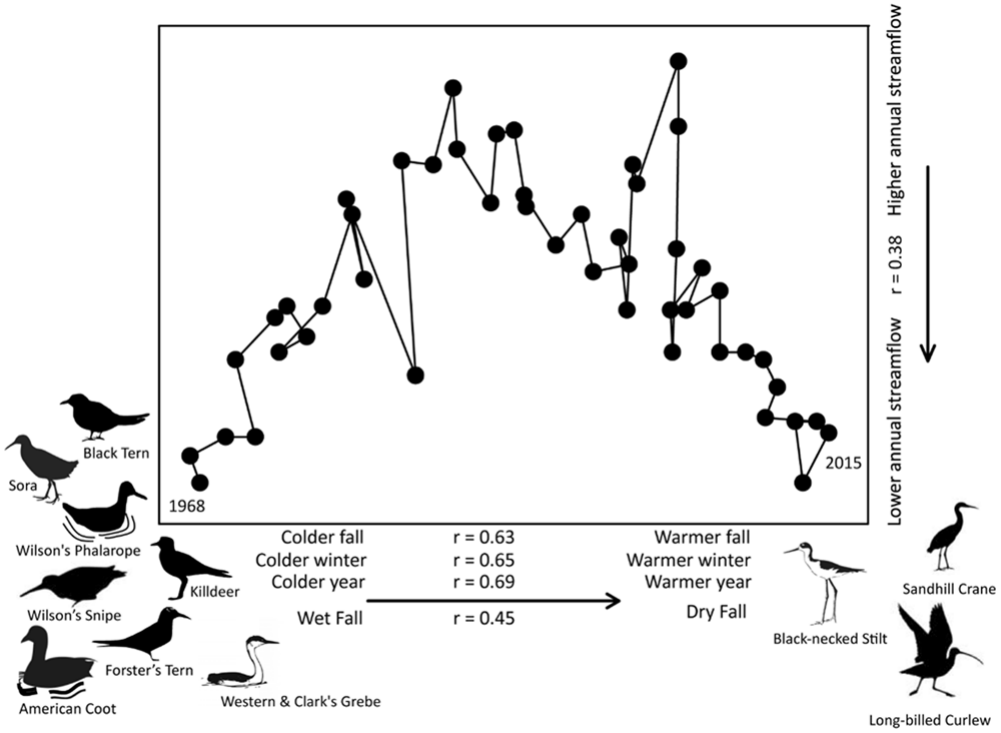
Changes in Great Basin breeding waterbird community composition and their association with climate over time (1968–2015; stress = 0.03) as indicated by a non-metric multi-dimensional scaling ordination plot. Circles represent the community composition each year whereas the distance between circles indicates their degree of similarity (higher proximity of circles indicates a higher similarity, whereas years that are more dissimilar are placed further apart). We include 1968 and 2015 as reference years to show direction of the community trajectory over time. Arrows located outside of each axis show the direction of statistically significant associations (*P* < 0.01) between climatic descriptors and each ordination axis score (1980–2015). Bird silhouettes illustrate species that showed statistically significant positive (right) and negative (left) trends in abundance over time as well as individual positive (right) and negative (left) associations to warm and dry climates (1968–2015; Supplementary Table S2). The humped shape of the curve illustrates changes in the community composition associated to streamflow (see secondary right axis). All species considered in this analysis migrate out of the Great Basin region, except for the American Coot, Wilson’s Snipe and Killdeer.

Our findings show remarkable changes over time in the composition of Great Basin waterbird species during late spring early summer. BBS data^45^ indicated that breeding abundances for 14 (of 25) Great Basin waterbird species met our selection criteria for analysis (see methods section; Fig. 4; Supplementary Table S2), and subsequently we found breeding waterbird species composition and abundances were linked to recent Great Basin climatic conditions (1980–2015). We detected statistically significant correlations (r ≥ |0.50|; *P* < 0.05) with at least one hydro-climatic variable for 11/14 species from 1980–2015. In most of the cases, a seasonal temperature metric was statistically significant. We found statistically significant associations between abundance indices for one waterbird species with spring mean air temperature, seven with summer, one with fall, and none with winter or annual mean temperature. Six species also were correlated with fall precipitation, and one species was positively associated with a 273-day stream magnitude. In addition, associations between long-term and large-scale trends in climate and hydrology for waterbird assemblages were evident from a non-metric multidimensional scaling analysis (nMDS; see methods section; Fig. 4). Finally, among eight open-water and shoreline foraging species that have undergone significant populations declines, five were negatively associated with temperature increases (Fig. 4; Supplementary Table S2): Killdeer (*Charadrius vociferus*), Wilson’s Snipe (*Gallinago delicata*), Black Tern (*Chlidonias niger*) and Western and Clark’s Grebe (*Aechmophorus occidentalis* and *A*. *clarkii*).

## Discussion

Here, we provide evidence that recent climate change is affecting an entire landscape of terrestrial and wetland ecosystems in a complex manner: climate-induced shifts in water volume drive changes in wetland salinity, which determines wetland community composition, which in turn determines waterbird use of the region. As the mix of wetlands shifts towards more saline and fewer freshwater wetlands, waterbirds experience increasing water stress. Together, these factors result in degradation of a continental migration route and important breeding area.

Increasing air temperature and a decrease in precipitation amount and timing has occurred across North America’s Great Basin over the past century, with a more recent and accelerating shift toward a warmer, drier climate. This is likely to produce and retain increasingly less snow in winter, lowering and shortening spring peak flows and habitat extent, which leads to longer, drier, and more saline summer-autumn conditions^32^. Thus, an increasing proportion and decreasing availability of saline and hypersaline aquatic communities leave fewer freshwater resources and other habitat choices for waterbirds throughout the annual cycle and across their Pacific Flyway migratory pathway. These landscape-scale changes in environmental water quantity and quality are consistent with recent studies documenting early timing of flow events in western North American rivers^35,36^ and are likely to continue given current climate-driven trends in temperature and precipitation regimes across the Great Basin and beyond^51^.

Inland arid land wetlands are poorly studied, and their relationship with climate and hydrologic change is complex. One challenge is finding appropriate datasets to examine these issues at adequate spatial and temporal scales. Here, we assumed that dynamics for Great Basin wetlands were primarily driven by local temperature and precipitation (i.e., higher temperatures imply drying wetlands). Certainly, additional wetland drivers (e.g., subsurface geology, groundwater hydrology, climate oscillations, and human water inputs, diversions, and withdrawals) play significant roles, but region-wide data are not available. Regional climate engines such as the Pacific Decadal Oscillation (PDO) and the El Niño–Southern Oscillation (ENSO) influence hydrology over a span of years but do not alter long-term observed trends^52^. We limit some of these additional complexities by using streamflow data from sites with relatively low human population density and focusing on a region that arguably may have simpler relationships between local climate and wetland and streamflow dynamics. Even so, the combination of these drivers may result in more complex, idiosyncratic, and often counterintuitive responses than simple assumptions about relationships. Future work could include site-specific modeling combined with monitoring hydrological and ecological variables to explore implications for resource management decision making and further test the climatic assumptions within this paper.

The pace of climate change in the Great Basin is similar to global measures, and the impact on this water-limited ecosystem is transforming the Great Basin and many ecological processes, including many if not most Great Basin species^22^. In the arid West, up to 60% of the observed hydrologic changes between 1950 and 1999, including reduced river flow and snowpack, are the result of human-caused climate change^32^. Drought conditions in the early 2000s that covered more than half the contiguous U.S. are a preview of future conditions that alter the seasonality and even existence of many wetlands^53^. Drier conditions will exacerbate the effects of human modifications at saline lakes, with additional decreases in streamflow and groundwater recharge^25,54^. Across the western U.S., snowpack is projected to decline by 60% in the next 30 years, driven especially by shifts in air temperature. Precipitation is strongly seasonal, with dry-season flows fed by winter snowpack. With warmer winter and spring air temperatures, less snow and more rain falls. As a result, less snow accumulates, so that the snowpack melt advances earlier in the season. Therefore, summers and autumns are projected to continue to grow longer and drier, effecting soil moisture, groundwater, and of course surface waters^37^.

Isolating the climate impacts on Great Basin waterbird population declines is challenging, yet these declines are fracturing a key step in the Pacific Flyway. The climate signal can be diffused or confounded by other influences. For example, water is often diverted from lakes and wetlands for cities and ranching, which results in less water to habitats already stressed by precipitation shifts. This complex interaction between anthropogenic actions and changing climate on wetland and waterbird viability can be seen at the Great Salt Lake where the freshwater inflows have been reduced by 39% over the past 150 years, leading to a 48% reduction in lake volume^53^. Drought conditions in the early 2000s resulted in a 33% decline in use by waterfowl when compared to wetter conditions^40,43^.

The combination of drought and diversion at hypersaline Lake Abert, Oregon, has resulted in recent inflows of less than half of what they have been compared to the historical recorded mean. In 2014–15, the lake declined from historic levels of more than 15,000 ha to 236 and 666 ha, respectively^29^. Between 2011 and 2015, counts of phalarope species went from more than 60,000 to less than 13,000 birds, and Eared Grebes from more than 11,000 to less than 100 birds^41^. Senner and colleagues^23^ found that as the area of Lake Abert decreased and salinity increased, invertebrate and waterbird numbers declined. Given the need for saline conditions, however, they also found that at high lake levels and low salinities, the abundance of most waterbirds and invertebrates either plateaued or declined. Thus, there is a small tolerance range for successful production of the invertebrate *Ephydra hians*, which is a critical food resource for adult waterbirds^55,56^.

Diversions from the Truckee River have caused water levels at Pyramid Lake, Nevada, to decline by almost 70 vertical feet since 1890^57^. White Pelican productivity is negatively correlated with the flow volume of the Truckee Canal, where flows have been diverted for agriculture since 1906^58^. Walker Lake in southwestern Nevada has dropped 150 vertical feet (75% reduction in volume) in the past century and salinity has increased 10-fold^59^. The salinity level is lethal to Lahontan Cutthroat trout (*Oncorhynchus clarkii henshawi*) and Tui chub (*Siphateles bicolor*) are declining, leaving a disappearing piscivorous food source (e.g., Common Loons, *Gavia immer*, are no longer seen on the lake). Nearby, diversions along the Carson River have led to an 84% loss of Lahontan Valley wetlands. Similar trends also have been observed just outside of the Great Basin including nearly 90% loss of wetlands in the Klamath Basin in southern Oregon and inflows at the Salton Sea in southern California dropped by 40% in 2018 with loss of water diverted from the Colorado River^22^.

Just as site degradation and loss reduces a migratory pathway, the impact of climate change may ultimately depend on synchronization of invertebrate productivity with waterbird movement patterns and the quantity and quality of wetland water resources available at the time. However, phenological mismatch might have the same outcome given that avian sex and age classes can differ in migration timing^60^. In theory, just as early breeding species may benefit from warmer winters and springs, so too would spring migrating waterbirds. Conversely, warmer environments in spring and summer can limit foraging options for chicks and adults as the number, quality and extent of water bodies decline. For example, Killdeer have restricted home ranges and depend primarily on freshwater sites throughout the year^61^. This renders them particularly susceptible to a warmer, drier climate as fewer freshwater outlets can decrease productivity of chicks that cannot metabolize salty water^46^.

A shortened hydroperiod also would negatively affect species such American Avocet that tend to remain in the Great Basin for months post-breeding^11,12^. Thus, in Great Basin aquatic systems, salinity drives productivity, and sites with freshwater and/or high salinity are optimally productive with respect to foraging needs of habitat specialists^62^. When salinity elevates beyond the optimal range, invertebrate prey experience a reduction in body size and fecundity, thus resulting in reduced foraging efficiency for avian predators^23,63–65^.

While we present waterbird species responding negatively as well as positively to a warmer, drier Great Basin, ultimately all species will continue to be stressed by an uninterrupted climate shifts. Climate models provide unclear projections for precipitation patterns, but even if winter precipitation increases, models concur around ongoing air temperature increases, exacerbating the long-term shift from a snowpack-dominated hydrology to a rain-fed hydrology, with longer and drier summers and autumns in the western U.S.^38,53,66,67^. Therefore, significant shifts in water management strategies are needed to buffer impacts on aquatic ecosystems in dry landscapes. On a local or regional scale, water management practices often involve upstream allocations and diversions that result in less water reaching terminal lakes and major wetlands, thereby accentuating water quality trends towards more saline and hypersaline wetlands^29^. Mitigating this pattern will require careful negotiation among upstream users. More water (such as the use of environmental flow regimes) may allow waterbird species to buffer against some impacts in coming decades, such as extreme droughts. More active resource management of Great Basin wetlands at a landscape scale — maintaining the mix of freshwater, saline, and hypersaline — may be a new option as well. For long-distance migratory waterbirds, coordinated regional, national and international resource management takes on new importance. Resource managers may even need to consider how and when to facilitate migratory pathways beyond established routes through concerted preparation of critical water resources^3^. Impacts are already clear enough to identify where water-related bottlenecks in migration connectivity may be occurring now or in the near future, suggesting water management strategies at the sites or nearby that may provide an impact mitigation strategy prior to system collapse.

The Great Basin represents significant habitat for millions of waterbirds during all phases of the annual cycle, hence, decreased availability of freshwater and decreased viability of perennial, hypersaline systems present significant risks for continuation of a major migratory route. Even loss of a small amount of habitat, food resource or a key site in this critical region could trigger disproportionate population declines, particularly because nearby options are becoming limited. Historically, wetlands in dry ecosystems have demonstrated great resilience and will continue to change. Already we have shown some waterbirds may benefit from the earlier onset of spring conditions. However, predicted climatic conditions, limited options for movement elsewhere, and similar results found for other inland migration routes suggests a serious scenario for migratory birds and others dependent on western wetlands.

## Methods

### Site description

The hydrographic Great Basin, the largest endorheic system in North America, covers nearly 440,000 km^2^ and portions of six states (California, Idaho, Nevada, Oregon, Utah and Wyoming; Fig. 1). The region is bound by the Sierra Nevada and Cascade Ranges to the west, the Wasatch Range and Colorado Plateau to the east, the Columbia Plateau to the north and the Mojave Desert to the south. In this internally draining system, precipitation generally falls as snow, and therefore, aquatic systems rely on spring recharge from snowmelt^32^. The water travels down from higher elevations and empties into low-lying, saline lakes or evaporates, thus creating a mosaic of aquatic systems with increasing wetland salinity. The unique drainage of the Great Basin creates a landscape in which water is disproportionately concentrated at the remnant waters. The Great Basin has a dry climate ranging from arid to semi-arid, but climate regimes during the Pleistocene and Holocene experienced enormous swings in temperature and precipitation^27–29^. The current saline and hypersaline systems were orders of magnitude larger as recently as a few thousand years ago and characterized by freshwater lentic and lotic communities. Currently, they are primarily lentic and very specialized for alkaline conditions.

### Temperature and precipitation

We investigated long-term seasonal and annual changes in air temperature and precipitation across the Great Basin using spatially explicit Parameter-elevation Regressions on Independent Slopes Model (PRISM) data. PRISM data provide for trend analyses at regional scales^35,51,68^, hence we used high-quality, spatially gridded, monthly minimum surface air temperatures and accumulated precipitation data generated from the PRISM dataset. The PRISM method interpolates available monthly temperature and precipitation observations from as many of the station networks and data sources as possible using a climatologically aided interpolation scheme that specifically accounts for the variation in station density over time. The PRISM-gridded climate data products recently have become a significant resource in ecology, biology, and other climate-related research^28^. PRISM data used in this study for long-term trend analysis were gridded monthly across the hydrographic Great Basin at 30-arcseconds (800 m^2^) from 1900–2008.

We averaged monthly minimum air temperatures across the water year (1 October-30 September) and across seasons (October-December, January-March, April-June, July-September) for each 800-m^2^ cell (n = 684,541). Although maximum and minimum air temperatures are changing, increases in minimum air temperatures are increasing at a faster rate in western North America^36^. Gridded maximum air temperature biases tend to be greater, while minimal air temperatures are influenced by large-scale climate conditions and may be relevant to ecosystem applications^69^.

### Streamflow changes

The hydrographic Great Basin contains 36-gauge stations designated as part of the USGS Hydro-Climatic Data Network (HCDN)^24^. Records began as early as 1905, although the majority were initiated later (Fig. 1, Supplementary Table S3) so the period of record for our study was 1920–2015. We analyzed trends in streamflow amounts by calculating the magnitude of the anomaly, defined as the accumulated 273-day (1 October-30 June) streamflow. We restricted the number of days to describing the magnitude of anomaly because of concerns over the amount of water available to breeding waterbirds in a given year. We included population indices of avian counts during the breeding season that were conducted during a two-week window each June (USGS Breeding Bird Survey, BBS; see below).

### Climate seasonality

We used the center of mass of annual flow, also known as the center of timing (CT), to assess changes in streamflow seasonality associated with shifts in climate for western North America^68,70^. For a given streamflow gauge, the CT represents the day of the water year in which 50% of the total annual flow has occurred. At each gauge station, we captured long-term changes to the seasonality of streamflow not only using the CT, but also by partitioning timing events into several percentiles (5^th^, 25^th^, 50^th^, 75^th^ and 95^th^) of the total annual flow. Stewart *et al*.^37^ calculated CT across HCDN streamflow gauges in North America to detect changes to the spring pulse in snowmelt dominated streams as:

1 $${\rm{CT}}={\rm{\Sigma }}({t}_{i}{q}_{i})/{\rm{\Sigma }}{q}_{i}$$

where *t*_*i*_ is time in days from the start of the water year and *q*_*i*_ is streamflow for days of the water year *i*.

### Statistical analyses

We computed long-term climatic trends using average monthly temperature and precipitation estimates that included seven metrics accounting for magnitude and timing of streamflow by season: Fall (October-December), Winter (January-March), Spring (April-June), Summer (July-September) and water year (Supplementary Table S4). All long-term climate summaries included the 5% and 95% ranges. We investigated trends from the corresponding time period for the BBS (1968–2015)^45^, and determined the significance of past trends for each climate and streamflow metric at each site using a non-parametric Mann-Kendall *tau* test for monotonic time series^49,50^. This rank-based test is robust for non-normal data, series with outliers and non-linear trends. When trends were significant (p ≤ 0.05), we estimated the magnitude of trends using the non-parametric Sen slope^71^. We used a block-bootstrap method to account for potential serial correlation effects^71^ and calculated trends for all metrics at different periods (1900–2008, 1920–2008, 1940–2008, 1960–2008, 1980–2008; Supplementary Table S5). We identified trends as detectable when the absolute values of their signal-to-noise ratios (SNRs) were greater than one. SNRs were calculated as the signal (trend slope multiplied by the period of record) divided by the standard deviation. We performed these statistical analyses using the software *R* ver. 2.11.1.

### Streamflow timing and seasonality

The flow regime also reflects climate patterns and has been referred to as the “master variable” of freshwater ecosystems^35^. To investigate the relationship between changing climates and flow regime, we examined long-term (1920–2015) trends using HCDN data^24^. Similar to climate trends, the magnitude of the anomaly of annual streamflow (1 October-30 June) indicated pronounced changes during the most contemporary period (1980–2015; Supplementary Table S1).

### Avian distribution

We used the USGS BBS indices of species abundance^45^ to better understand how changes in climate flow regime may be influencing Great Basin waterbird distributions and habitat use over time. Initiated in the western U.S. in 1968, the BBS is a long-term monitoring program that tracks the abundance and distribution of breeding birds in North America. Data are collected during peak breeding season (typically late May and June) through roadside surveys along 40-km long routes. Observers conduct three-minute point counts every 800 m along each survey route. Sauer *et al*.^45^ employed a hierarchical log-linear model to analyze BBS data and describe avian population change information by species and region using a scaled index of abundance. We used an updated PRISM climate data through 2015 to investigate impacts on avian species abundance trends and assemblages. We considered only waterbird species predominantly associated with lakes and wetlands known to breed abundantly throughout the Great Basin (Supplementary Table S2). We excluded waterfowl from our analyses so as to emphasize the situation for under-represented waterbirds. However, waterfowl face similar issues related to climate change in the Great Basin^9,22,43^.

Estimating regional population trends from BBS data introduces a variety of potential problems that may bias the results. Therefore, BBS data were categorized into three levels of credibility: low, moderate and high. Records were assigned a credibility level based on sample size, low abundance on survey routes, imprecise trends and absent data. Thus, we evaluated only species identified as “moderate” or “high” credibility from 1968–2015 for the Great Basin physiographic area.

Changes in breeding bird assemblages were evaluated based on the similarity among years using the Bray-Curtis index of similarity^32^ and visualized using an nMDS unconstrained ordination technique. The Bray-Curtis similarity resemblance matrix was created using the square root transformed BBS index^45^. The nMDS technique places each year in a multivariate space in the most parsimonious arrangement (relative to each other) with no *a priori* hypotheses. Based on an iterative optimization procedure (999 random starts), we minimized a measure of disagreement or stress between their distances in 2-D^72^. It is suggested^32^ that a stress value lower than 0.05 represent an excellent ordination, stress <0.1 is a good ordination, stress <0.2 an acceptable ordination, and stress >0.2 a poor ordination. The resulting scores from the 2-D plot provided a collective index of how unique the bird assemblage was in a given year. Higher proximity of points in the 2-D plot indicated a higher similarity, whereas more dissimilar points were placed further apart. Lastly, we used a Pearson’s correlation coefficient to examine strength of the association between scores from the 2-D ordination plot and several annual hydro-climate descriptors (Supplementary Table S4). We repeated this procedure to examine the strength of the association between climate descriptors and BBS indices for individual species. We displayed the strongest correlations (r ≥ |0.30|) in the 2-D plot.

## Supplementary information

Supplementary Information

## Data Availability

The datasets generated during and/or analyzed during this study are included in this published article and its Supplementary Information.
