# Supplementary material for: Climate-Altered Wetlands Challenge Waterbird Use and Migratory Connectivity in Arid Landscapes
Source: Sci Rep. 2019 Mar 15;9:4666. doi: 10.1038/s41598-019-41135-y (PMC6420639; doi:10.1038/s41598-019-41135-y)
Supplement: Supplementary file 1 — Supplementary Information [file 41598_2019_41135_MOESM1_ESM.docx]

**Supplementary Information**

Climate-Altered Wetlands Challenge Waterbird Use and Migratory Connectivity

in Arid Landscapes

*Susan M. Haig^1^, Sean P. Murphy^1,2^, John H. Matthews^3^, Ivan Arismendi^4^, Mohammad Safeeq^5^

^1^U.S. Geological Survey, Forest and Rangeland Ecosystem Science Center, Corvallis, Oregon, USA.

^2^Current address: Pennsylvania Game Commission, Harrisburg, Pennsylvania, USA.

^3^Alliance for Global Water Adaptation, Corvallis, Oregon, USA.

^4^Department of Fisheries and Wildlife, Oregon State University, Corvallis, Oregon, USA.

^5^Sierra Nevada Research Institute, University of California-Merced, Merced, California, USA.

*Corresponding author: [susan_haig@usgs.gov](mailto:susan_haig@usgs.gov); 541-760-9151

**
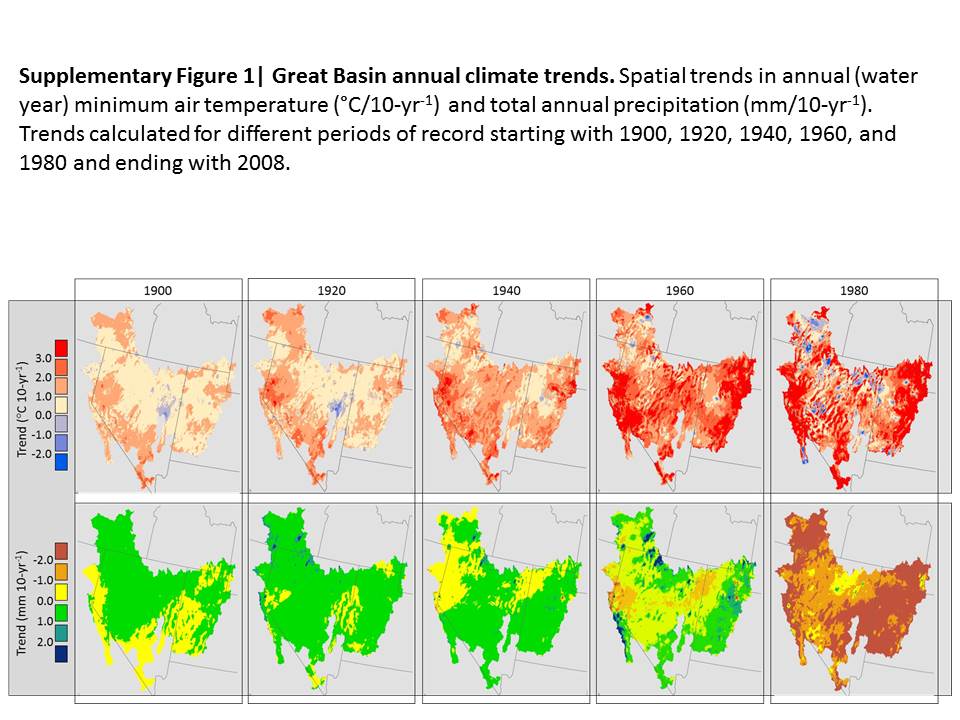
**

**Supplementary Figure S1.** Great Basin annual climate trends 1900-2008. Spatial distribution of trends in annual (water year) minimum air temperature (°C/decade) and total annual precipitation (mm/decade). Trends calculated for 1900-2008, 1920-2008, 1940-2008, 1960-2008 and 1980-2008.


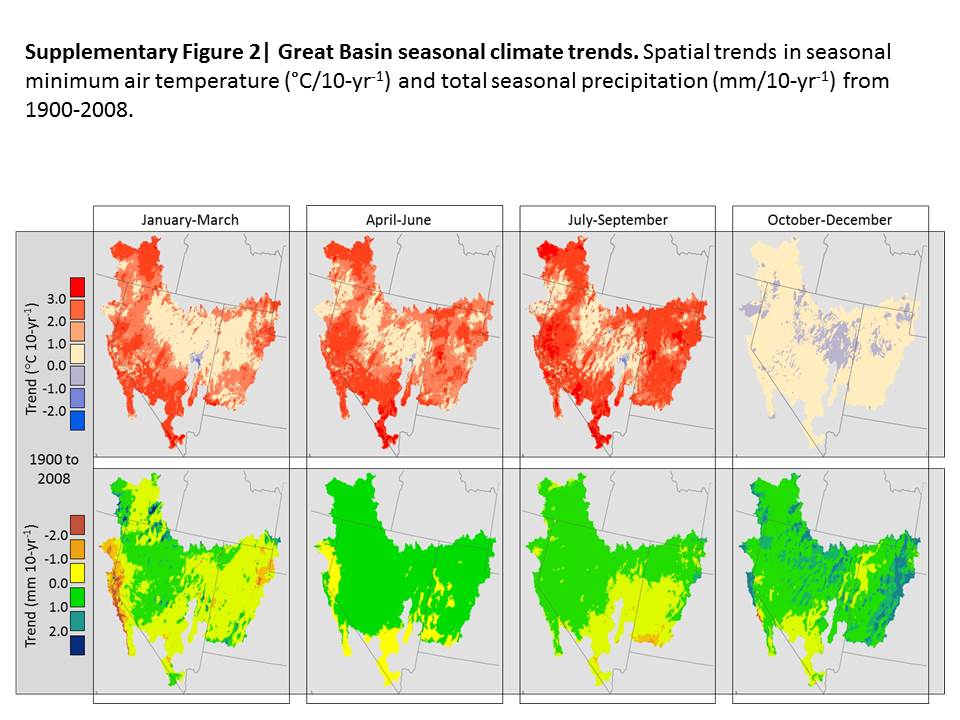


**Supplementary Figure S2.** Great Basin seasonal climate trends**.** Spatial distribution of trends in seasonal minimum air temperature (°C/decade) and total seasonal precipitation (mm/decade) from 1900-2008.

**
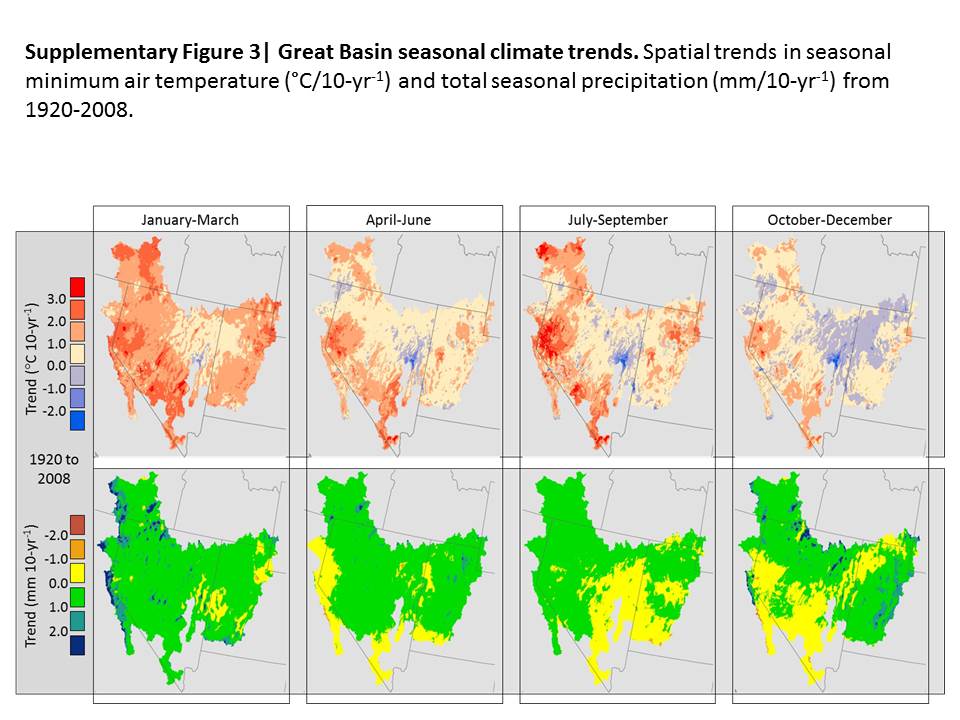
**

**Supplementary Figure S3.** Great Basin seasonal climate trends. Spatial distribution of trends in seasonal minimum air temperature (°C/decade) and total seasonal precipitation (mm/decade) from 1920-2008.


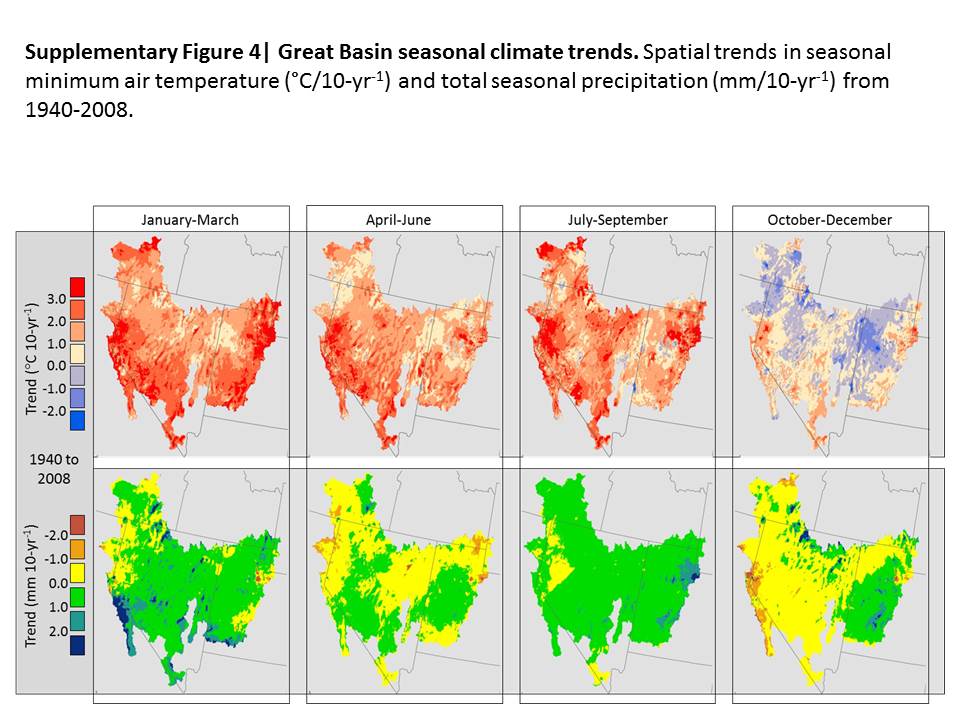


**Supplementary Figure S4.** Great Basin seasonal climate trends. Spatial distribution of trends in seasonal minimum air temperature (°C/decade) and total seasonal precipitation (mm/decade) from 1940-2008.


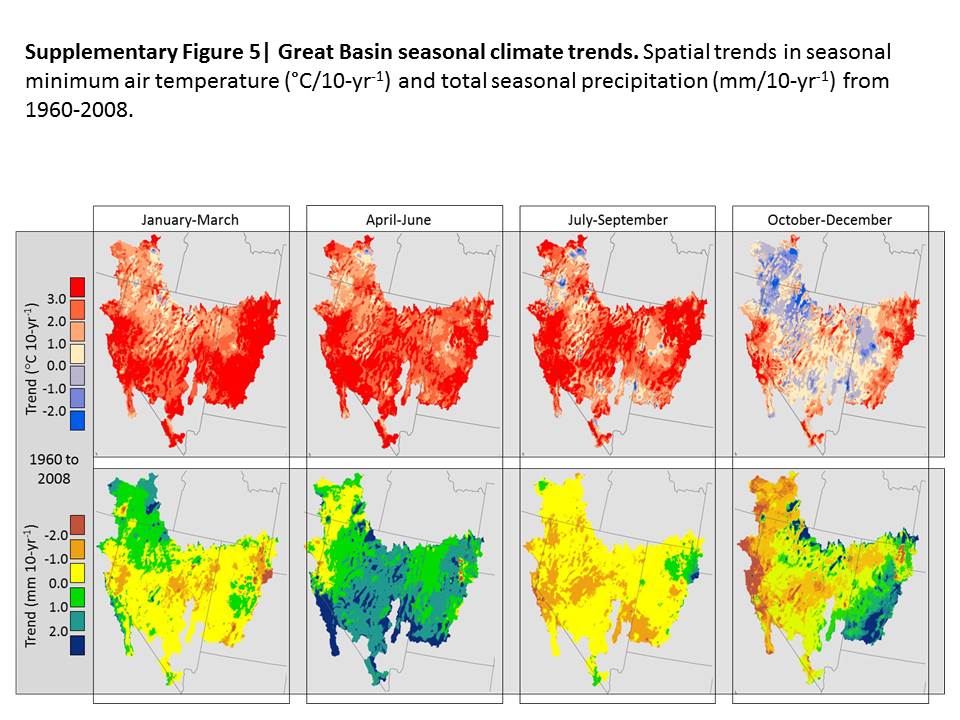


**Supplementary Figure S5.** Great Basin seasonal climate trends**.** Spatial distribution of trends in seasonal minimum air temperature (°C/decade) and total seasonal precipitation (mm/decade) from 1960-2008.


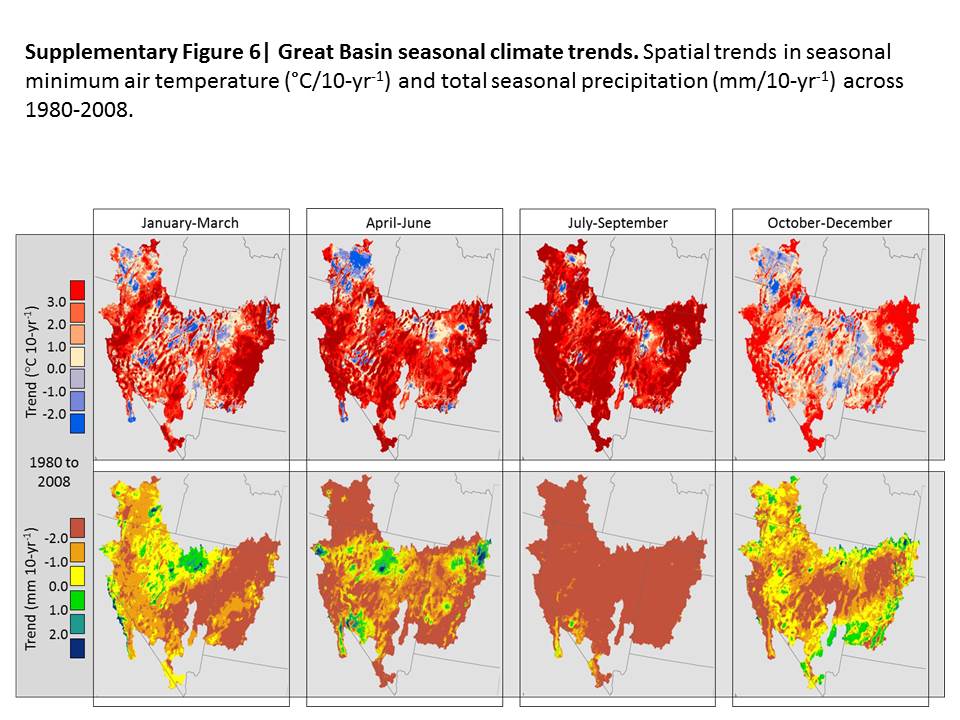


**Supplementary Figure S6.** Great Basin seasonal climate trends. Spatial distribution of trends in seasonal minimum air temperature (°C/decade) and total seasonal precipitation (mm/decade) from 1980-2008.

**Supplementary Table S1.** Changes in timing (including five percentiles), duration and magnitude of Great Basin streamflow events (1980-2015). Magnitude of trends in timing of the annual percentile flow (days every 30 years) among Great Basin streams (n = 29). Positive values indicate later arrival of streamflow events whereas negative values indicate earlier arrival of streamflow events. Magnitude of trends in the duration of the hydroperiod (days every 30 years) and annual streamflow (streamflow anomaly every 30 years) are also included. Magnitude of significant trends using the regional Mann-Kendall test are shown in parentheses.

|  | Magnitude of trends in timing of percentile  of annual flow (days / 30 years) | | | | |  |  |
| --- | --- | --- | --- | --- | --- | --- | --- |
|  | 5^th^ | 25^th^ | Magnitude of anomaly in annual streamflow^2^ | 75^th^ | 95^th^ | Magnitude of trends in the duration of hydroperiod^1^ | Magnitude of trends in annual streamflow anomaly^2^ |
| Mean | 1.0 | -0.6 | -0.8 (-2.7) | -2.1 (-3.0) | -2.2 | -3.2 | -0.8 (-0.8) |
| Maximum | 42.5 | 21.8 | 22.5 | 7.5 | 20.9 | 40 | -1.4 |
| Minimum | -20.0 | -30 | -20.6 | -12.4 | -25 | -57 | -0.4 |
| SD | 13.6 | 15 | 8.5 | 4.6 | 10.3 | 21.5 | 0.2 |
| ^1^days between the 5^th^ and 95^th^ percentile | | | | | | | |
| ^2^1 Oct-30 Jun | | | | | | | |

**Supplementary Table S2.** Trends in Great Basin waterbird breeding abundances (1968-2015) and their association with hydroclimate (1980-2015). Correlations between the index of annual bird abundances and seasonal (winter = January-March; spring = April-June; summer = July-September; fall = October-December) and annual conditions of temperature and precipitation. Values ≥ |0.50| in **bold**.

|  | Trends in waterbird abundances via USGS BBS^†^ (1968-2015) | |  | Associations between bird abundance and temperature (1980-2015) | | | | |
| --- | --- | --- | --- | --- | --- | --- | --- | --- |
| Species | Trend^††^ | *P-value*^†††^ |  | Winter | Spring | Summer | Fall | Annual |
| American Avocet (*Recurvirostra americana*) | -1.32 | 0.024 |  | 0.20 | 0.16 | -0.23 | **0.57** | 0.48 |
| **American Coot (*Fulica americana*)** | -1.77 | **0.000** |  | 0.09 | 0.39 | **-0.51** | 0.17 | 0.14 |
| **Black Tern (*Chlidonias niger*)** | -2.43 | **0.000** |  | 0.04 | 0.34 | **-0.62** | 0.26 | 0.28 |
| **Black-necked Stilt (*Himantopus himantopus*)** | 0.65 | **0.000** |  | 0.13 | -0.36 | **0.56** | 0.22 | 0.13 |
| **Forster's Tern (*Sterna forsteri*)** | -0.92 | **0.000** |  | 0.12 | 0.29 | -0.48 | 0.17 | 0.25 |
| **Killdeer (*Charadrius vociferus*)** | -3.29 | **0.000** |  | 0.02 | 0.48 | -0.48 | 0.05 | 0.12 |
| **Long-billed Curlew (*Numenius americanus*)** | 1.35 | **0.000** |  | 0.01 | -0.28 | **0.67** | -0.15 | -0.15 |
| **Sandhill Crane (*Grus canadensis*)** | 1.23 | **0.000** |  | 0.05 | -0.33 | **0.68** | 0.03 | -0.03 |
| **Sora (*Porzana carolina*)** | -0.03 | **0.000** |  | 0.02 | **0.51** | -0.38 | 0.24 | 0.06 |
| Spotted Sandpiper (*Actitis macularius*) | 0.01 | 0.120 |  | 0.19 | -0.10 | 0.08 | 0.31 | 0.25 |
| **Western/Clark's Grebe (*Aechmophorus occidentalis/A. clarkii*)** | -3.18 | **0.001** |  | 0.10 | 0.34 | **-0.54** | 0.35 | 0.36 |
| Willet (*Tringa semipalmata*) | -0.07 | 0.298 |  | 0.04 | 0.41 | 0.35 | -0.06 | -0.20 |
| **Wilson's Phalarope (*Phalaropus tricolor*)** | -0.42 | **0.000** |  | 0.30 | 0.03 | -0.42 | 0.02 | 0.24 |
| **Wilson's Snipe (*Gallinago delicata*)** | -1.29 | **0.000** |  | 0.02 | 0.45 | **-0.62** | 0.03 | 0.06 |
| ^†^ U.S. Geological Survey Breeding Bird Survey (Sauer et al. 2017).  ^††^ Magnitude (Sen slope every 30 years) of trend by single species. | | | | | | | | |
| ^†††^ Corrected *P-value* (*P < 0.01 in bold*) accounting for potential serial correlation effects^63^.  **Supplementary Table S2 cont.** | | | | | | | | |
|  | Trends in waterbird abundances via USGS BBS^†^ (1968-2015) | |  | Associations between bird abundance and precipitation (1980-2015) | | | | |
| Species | Trend^††^ | *P-value*^†††^ |  | Winter | Spring | Summer | Fall | Annual |
| American Avocet (*Recurvirostra americana*) | -1.32 | 0.024 |  | 0.27 | -0.10 | -0.28 | **0.77** | 0.08 |
| **American Coot (*Fulica americana*)** | -1.77 | **0.000** |  | 0.29 | 0.12 | -0.08 | **0.52** | 0.23 |
| **Black Tern (*Chlidonias niger*)** | -2.43 | **0.000** |  | 0.42 | 0.04 | -0.24 | **0.57** | 0.22 |
| **Black-necked Stilt (*Himantopus himantopus*)** | 0.65 | **0.000** |  | -0.26 | -0.26 | 0.04 | -0.09 | -0.31 |
| **Forster's Tern (*Sterna forsteri*)** | -0.92 | **0.000** |  | 0.40 | -0.06 | -0.22 | **0.50** | 0.17 |
| **Killdeer (*Charadrius vociferus*)** | -3.29 | **0.000** |  | 0.46 | 0.12 | -0.12 | 0.44 | 0.34 |
| **Long-billed Curlew (*Numenius americanus*)** | 1.35 | **0.000** |  | -0.27 | -0.14 | 0.16 | -0.41 | -0.19 |
| **Sandhill Crane (*Grus canadensis*)** | 1.23 | **0.000** |  | -0.25 | -0.20 | 0.08 | -0.32 | -0.23 |
| **Sora (*Porzana carolina*)** | -0.03 | **0.000** |  | 0.35 | 0.19 | 0.10 | 0.45 | 0.36 |
| Spotted Sandpiper (*Actitis macularius*) | 0.01 | 0.120 |  | 0.04 | -0.18 | -0.18 | **0.56** | -0.06 |
| **Western/Clark's Grebe (*Aechmophorus occidentalis, A. clarkii*)** | -3.18 | **0.001** |  | 0.47 | -0.03 | -0.28 | **0.63** | 0.21 |
| Willet (*Tringa semipalmata*) | -0.07 | 0.298 |  | 0.18 | 0.15 | 0.19 | -0.10 | 0.28 |
| **Wilson's Phalarope (*Phalaropus tricolor*)** | -0.42 | **0.000** |  | -0.03 | 0.01 | -0.33 | 0.39 | -0.09 |
| **Wilson's Snipe (*Gallinago delicata*)** | -1.29 | **0.000** |  | 0.38 | 0.17 | -0.11 | 0.44 | 0.32 |

**Supplementary Table S2 cont.**

|  | Trends in waterbird abundances USGS BBS^†^ (1968-2015) | |  | Associations between bird abundance and streamflow (1980-2015) | |
| --- | --- | --- | --- | --- | --- |
| Species | Trend^††^ | *P-value*^†††^ |  | Duration | Magnitude |
| American Avocet (*Recurvirostra americana*) | -1.32 | **0.024** |  | 0.20 | 0.16 |
| **American Coot (*Fulica americana*)** | -1.77 | **0.000** |  | 0.09 | 0.39 |
| **Black Tern (*Chlidonias niger*)** | -2.43 | **0.000** |  | 0.04 | 0.34 |
| **Black-necked Stilt (*Himantopus himantopus*)** | 0.65 | **0.000** |  | 0.13 | -0.36 |
| **Forster's Tern (*Sterna forsteri*)** | -0.92 | **0.000** |  | 0.12 | 0.29 |
| **Killdeer (*Charadrius vociferus*)** | -3.29 | **0.000** |  | 0.02 | 0.48 |
| **Long-billed Curlew (*Numenius americanus*)** | 1.35 | **0.000** |  | 0.01 | -0.28 |
| **Sandhill Crane (*Grus canadensis*)** | 1.23 | **0.000** |  | 0.05 | -0.33 |
| **Sora (*Porzana carolina*)** | -0.03 | **0.000** |  | 0.02 | **0.51** |
| Spotted Sandpiper (*Actitis macularius*) | 0.01 | 0.120 |  | 0.19 | -0.10 |
| **Western/Clark's Grebe (*Aechmophorus occidentalis/A. clarkii*)** | -3.18 | **0.001** |  | 0.10 | 0.34 |
| Willet (*Tringa semipalmata*) | -0.07 | 0.298 |  | 0.04 | 0.41 |
| **Wilson's Phalarope (*Phalaropus tricolor*)** | -0.42 | **0.000** |  | 0.30 | 0.03 |
| **Wilson's Snipe (*Gallinago delicata*)** | -1.29 | **0.000** |  | 0.02 | 0.45 |

**Supplementary Table S3.** U.S. Geological Survey streamflow data set for Great Basin climate studies**.** The 36 Great Basin Hydro-Climatic Data Network (HCDN) stream gauges, station number, name, state located, year initiated, and years of record. We used a period of record from 1920-2015 to evaluate trends in streamflow hydroperiod.

| State | Stream | Station No. | Year 1 | N Years |
| --- | --- | --- | --- | --- |
| CA | Blackwood Creek | 10336660 | 1961 | 55 |
| CA | Carson R., East Fork | 10308200 | 1961 | 55 |
| CA | Carson R., West Fork | 10310000 | 1939 | 77 |
| CA | Sagehen Creek | 10343500 | 1954 | 62 |
| CA | Trout Creek | 10336780 | 1961 | 55 |
| CA | West Walker River 1 | 10296000 | 1939 | 77 |
| CA | West Walker River 2 | 10296500 | 1920 | 76 |
| ID | Cub River | 10093000 | 1941 | 48 |
| NV | Carson River | 10312000 | 1934 | 82 |
| NV | Chiatovich Creek | 10249900 | 1961 | 22 |
| NV | Humboldt River | 10322500 | 1920 | 96 |
| NV | Lamoille Creek | 10316500 | 1920 | 75 |
| NV | Martin Creek | 10329500 | 1922 | 94 |
| NV | McDermitt Creek | 10352500 | 1949 | 66 |
| NV | Quinn River | 10353500 | 1949 | 35 |
| NV | Reese River | 10325500 | 1952 | 29 |
| NV | South Twin River | 10249300 | 1966 | 50 |
| NV | Steptoe Creek | 10244950 | 1967 | 49 |
| NV | Walker River | 10301500 | 1945 | 71 |
| OR | Chewaucan River | 10384000 | 1925 | 91 |
| OR | Deep Creek | 10371500 | 1933 | 83 |
| OR | Donner River | 10396000 | 1939 | 77 |
| OR | Silvies River | 10393500 | 1924 | 83 |
| UT | Beaver River | 10234500 | 1920 | 96 |
| UT | Blacksmith Fork | 10113500 | 1920 | 92 |
| UT | Chalk Creek | 10131000 | 1928 | 88 |
| UT | Oak Creek | 10208500 | 1965 | 25 |
| UT | Provo R., North Fork | 10153800 | 1964 | 33 |
| UT | Red Butte Creek | 10172200 | 1964 | 52 |
| UT | Salina Creek | 10205030 | 1964 | 52 |
| UT | Sevier River | 10174500 | 1920 | 85 |
| UT | Trout Creek | 10172870 | 1960 | 49 |
| UT | Vernon Creek | 10172700 | 1959 | 57 |
| UT | Weber River | 10128500 | 1920 | 96 |
| WY  WY | Smiths Fork  Sulphur Creek | 10032000  10015700 | 1943  1958 | 73  40 |

**Supplementary Table S4.**  Hydro-climatic descriptors of Great Basin water flow**.** Measures of long-term annual changes in climate and hydrology in the Great Basin calculated to examine associations with Breeding Bird Survey indices. Climate measures are aggregate average anomalies.

| Measure | | Definition |
| --- | --- | --- |
| Hydrology | |  |
|  | 5^th^ flow | Day of year that 5% of the annual flow has occurred. |
|  | 25^th^ flow | Day of year that 25% of the annual flow has occurred. |
|  | 50^th^ flow | Day of year that 50% of the annual flow has occurred. |
|  | 75^th^ flow | Day of year that 75% of the annual flow has occurred. |
|  | 95^th^ flow | Day of year that 95% of the annual flow has occurred. |
|  | Annual average | Annual average calculated from monthly flows. |
|  | Center of timing | Timing of the center of mass of annual flow |
|  | Fractional flow | Snowmelt seasonal fractional flow; |
|  |  | ratio of streamflow April-July to total water year |
|  | Duration | Days from 5^th^ flow to 95^th^. |
|  | Magnitude | 273-day (1 Oct-30 Jun) anomalized flow magnitude. |
|  |  |  |
| Climate | |  |
|  | Winter temp. | Minimum Jan-Mar temperature. |
| Spring temp. | | Minimum Apr-Jun temperature. |
| Summer temp. | | Minimum Jul-Sep temperature. |
| Fall temp. | | Minimum Oct-Dec temperature. |
| Annual temp. | | Minimum annual temperature. |
| Winter precip. | | Jan-Mar monthly total precipitation (mm). |
| Spring precip. | | Apr-Jun monthly total precipitation (mm). |
| Summer precip. | | Jul-Sep monthly total precipitation (mm). |
| Fall precip. | | Oct-Dec monthly total precipitation (mm). |
| Annual precip. | | Average annual precipitation (mm). |

**Supplementary Table S5.** Proportion of detectable climate trends over 100 years in the Great Basin. The proportion of gridded climate cells (800-m^2^; *n* = 684,541) for each season and annually with a detectable trend [signal-to-noise ratio (SNR) > |1|]. The SNR was defined as the absolute change in the climate variable (temperature, precipitation) calculated from the trend divided by the standard deviation over the period of record (1900-2008, 1980-2008).

| Climate variable | Period | 1900-2008 | 1980-2008 |
| --- | --- | --- | --- |
| Temperature | Annual | 60.5 | 52.3 |
|  | Jan-Mar | 7.3 | 8.6 |
|  | Apr-Jun | 44.6 | 23.3 |
|  | Jul-Sep | 66.3 | 61.8 |
|  | Oct-Dec | 9.9 | 23.3 |
|  |  |  |  |
| Precipitation | Annual | 1.6 | 35.5 |
|  | Jan-Mar | 0.1 | 2.9 |
|  | Apr-Jun | 0.2 | 2.8 |
|  | Jul-Sep | 0.0 | 57.6 |
|  | Oct-Dec | 0.1 | 2.8 |
